# Sequence determinants of human microsatellite variability

**DOI:** 10.1186/1471-2164-10-612

**Published:** 2009-12-16

**Authors:** Trevor J Pemberton, Conner I Sandefur, Mattias Jakobsson, Noah A Rosenberg

**Affiliations:** 1 Department of Human Genetics, University of Michigan, 100 Washtenaw Avenue, Ann Arbor, Michigan 48109 USA; 2 Center for Computational Medicine and Biology, University of Michigan, 100 Washtenaw Avenue, Ann Arbor, Michigan 48109 USA; 3 Department of Evolutionary Biology, Evolutionary Biology Center, Uppsala University, Norbyvägen 18D, SE-752 36 Uppsala, Sweden

## Abstract

**Background:**

Microsatellite loci are frequently used in genomic studies of DNA sequence repeats and in population studies of genetic variability. To investigate the effect of sequence properties of microsatellites on their level of variability we have analyzed genotypes at 627 microsatellite loci in 1,048 worldwide individuals from the HGDP-CEPH cell line panel together with the DNA sequences of these microsatellites in the human RefSeq database.

**Results:**

Calibrating PCR fragment lengths in individual genotypes by using the RefSeq sequence enabled us to infer repeat number in the HGDP-CEPH dataset and to calculate the mean number of repeats (as opposed to the mean PCR fragment length), under the assumption that differences in PCR fragment length reflect differences in the numbers of repeats in the embedded repeat sequences. We find the mean and maximum numbers of repeats across individuals to be positively correlated with heterozygosity. The size and composition of the repeat unit of a microsatellite are also important factors in predicting heterozygosity, with tetra-nucleotide repeat units high in G/C content leading to higher heterozygosity. Finally, we find that microsatellites containing more separate sets of repeated motifs generally have higher heterozygosity.

**Conclusions:**

These results suggest that sequence properties of microsatellites have a significant impact in determining the features of human microsatellite variability.

## Background

Microsatellite loci consist of short tandem repeats (STR) that vary in length between individuals and that generally have many distinct alleles within a population. The high level of variability for microsatellites compared to other genomic regions [1-4] and their abundance in diverse genomes [5-9] have led to their use as markers in many settings, including linkage analysis [10-14], forensic investigations [15-21], human population genetics [22-27], and phylogeny reconstruction [28-30].

Microsatellites are among the fastest-evolving DNA sequences, with relatively high mutation rates of at least 10^-6^-10^-3 ^events per locus per gamete per generation, as measured in humans [2,31-36], other mammals [37-39], plants [40-42], and various other organisms [43-47]. It is this high mutation rate that has been a key factor in determining the informativeness of microsatellites in genetic studies. They have been used extensively over the last 10-15 years to investigate genetic variation across populations in many species [22,48-63].

The sequence properties of microsatellites have also been studied in a variety of organisms. Database analyses of genomic sequences have investigated the genome-wide frequency and distribution of microsatellites; such analyses find that the frequencies of microsatellites with different repeat motifs, as well as their average and maximum repeat lengths, vary widely both between and within motif size classes (di-, tri-, tetra-, or penta-nucleotide) [7,8,64-68]. Many microsatellites consist of uninterrupted, or "perfect", sets of consecutive repeats. However, a microsatellite can also be comprised of adjacent tandem arrays of different repeat motifs (termed "compound" [69]) or "interrupted" as a result of point mutations and small insertions or deletions that have occurred during the evolution of the locus (also termed "imperfect").

Whereas sequence studies of microsatellites rely on complete or partial genome sequences, typical microsatellite studies of genetic diversity instead use data sets of polymerase chain reaction (PCR) fragment lengths measured for microsatellite loci in a collection of individuals. Each fragment length is obtained using locus-specific DNA primer pairs to amplify the specific region of the genome containing a particular microsatellite. A PCR fragment length represents the size of the region between the distal ends of a DNA primer pair, with changes in the number of repeats of the microsatellite embedded between the primer pair leading to corresponding changes in PCR fragment length. Thus, differences in fragment length are used as a proxy for differences in the number of repeats. However, DNA primer pairs are placed to optimize their PCR amplification efficiency rather than to satisfy specific distance criteria, and the distances of primers from the embedded repeat sequence vary greatly between loci. Differences in PCR fragment lengths are therefore representative of differences in repeat number for genotypes of a given microsatellite locus, but they do not allow absolute numbers of repeats to be determined. The lengths of PCR fragments also do not provide information about other underlying sequence properties, such as base composition of repeat motifs, interruptions, and adjacency of separate tandem arrays.

In this study we aim to link diversity in human populations with underlying sequence properties for 627 microsatellite loci. Using the human RefSeq sequence for each locus, we investigate the effects of intrinsic genomic properties of microsatellites - namely the size and sequence of their repeat units, the number of separate STR regions embedded in their sequences and the distance separating such regions if more than one of them is found, and the properties of the sequences flanking the STR regions - on features of their genetic diversity in a worldwide sample of 1,048 individuals. Assuming that differences in PCR fragment length reflect differences in the numbers of repeats in the embedded STRs, we use the human RefSeq sequence to calibrate PCR fragment lengths in individual genotypes for inferring repeat numbers for the 627 microsatellite loci in the worldwide data set. This calibration enables us to investigate the effect of the mean, minimum, and maximum of the number of repeats across individuals on statistics measuring genetic diversity. Two previous reports on microsatellite loci in *Drosophila melanogaster *found heterozygosity to be positively correlated with the mean [70] and maximum [70,71] number of repeats; however, two other reports, also in *D. melanogaster*, did not find statistically significant correlations [72,73]. To the best of our knowledge, in humans, because of the limitations of PCR-based genotyping, the relationship of sequence properties of human microsatellites and properties of microsatellite genetic diversity has yet to be explored in detail.

## Methods

### Microsatellite genotype data

The data set that we analyzed consisted of 1,048 individuals from the HGDP-CEPH Human Genome Diversity Cell Line Panel [74] genotyped for 783 microsatellites spread across all 22 autosomes. These 783 microsatellites were comprised of the 377 loci from Marshfield Screening Set no. 10 that were previously reported by Rosenberg *et al*. [26], as well as the 406 additional loci from Marshfield Screening Sets no. 13 and 52 that were previously reported by Ramachandran *et al*. [75] and Rosenberg *et al*. [76]. For each microsatellite locus the genotype data consisted of PCR fragment lengths in each individual, obtained using locus-specific DNA primer pairs to amplify the specific region of the genome containing that microsatellite.

### Microsatellite primer sequences

Primer pairs for all 783 microsatellites were obtained from the publicly available primer sequence file provided by the Mammalian Genotyping Service [77] (Marshfield, WI) http://research.marshfieldclinic.org/genetics/GeneticResearch/screeningsets.asp for the Screening Set from which their genotypes were obtained (Screening Sets no. 10, 13, or 52), with seven exceptions. The primer pair for GTTTT002P was not present in the primer sequence file for Screening Set no. 13 from which its genotypes were obtained, but it was available for Screening Set no. 53. The primer pairs for MFD424-TTTA003, GATA23G09, AAT238, TTTA063, ATA008, and ATA43C09 were not present in the primer sequence file for Screening Set no. 52 from which their genotypes were obtained, but they were available for the preceding Screening Set no. 51. For each of these seven microsatellites, the chromosomal location and allele size range provided for the microsatellite in Marshfield Screening Set 53 or 51 matched that given in Screening Set 13 or 52, respectively. Primers for these seven microsatellites were therefore taken from these alternate Screening Sets as proxies for the desired Screening Sets.

Screening Set no. 10 was genotyped for the Rosenberg *et al*. [26] study prior to the genotyping of Screening Sets no. 13 and 52, with genotypes from the latter microsatellite sets being added to the original data [26] only for microsatellites not already genotyped in Screening Set no. 10. Therefore, in instances in which a microsatellite was present in both Screening Set no. 10 and Screening Set no. 13 or 52, primers were taken from Screening Set no. 10. The primer pairs used in this study can be found in Table S1 (Additional File 1).

### BLAST analysis of primers and extraction of sequences

Each forward primer sequence and each reverse primer sequence was separately used as the query sequence in BLASTN searches of the human genome RefSeq database [78] (release 28) using the standalone *blastall *application [79] (version 2.2.18) with the repetitive sequence filter turned off and the expected value (e) set to 1000. For each microsatellite locus, BLASTN "hits" that aligned along the entire length of the forward primer and that were on the correct chromosome for the locus were identified and were ranked by their e-value, from lowest to highest. Similarly, BLASTN "hits" that aligned along the entire length of the reverse primer and that were on the correct chromosome for the locus were also identified and were ranked by their e-value, from lowest to highest. The size of the fragment demarcated by the forward primer "hit" that had the lowest e-value and the reverse primer "hit" that had the lowest evalue was calculated as the distance between the terminal 5' nucleotide of the forward primer "hit" and the terminal 5' nucleotide of the reverse primer "hit". This fragment size was then compared against the allele size range provided by the Mammalian Genotyping Service [77] (henceforth "Marshfield") for the corresponding microsatellite locus and against the allele size range among individuals in the HGDP-CEPH data set. If the reverse primer used to genotype a microsatellite locus had been modified with a 6 bp pig-tail or with a single extra adenine base, then a one letter suffix, P or M respectively, was included in the Marshfield marker name. For those primer pairs for which the reverse primer was listed in the HGDP-CEPH data set as having been modified with a 6 bp pig-tail or with a single extra adenine base, the size of the fragment demarcated by the primer pair was adjusted by the addition of 6 bp or 1 bp, respectively, prior to comparison with the ranges expected. For a microsatellite to be flagged as "found" in the RefSeq database, the size of the fragment demarcated by the forward primer "hit" that had the lowest e-value and the reverse primer "hit" that had the lowest e-value had to meet one of three criteria. (i) First, it could be within both the allele size range provided by Marshfield and the allele size range computed from the HGDP-CEPH data set. (ii) If the fragment size was outside one or both of these ranges, then we calculated a quantity that we term *ROS*, for "range overlap score". If the smallest and largest allele sizes in the range provided by Marshfield are denoted by *m *and *M*, respectively, and the smallest and largest allele sizes in the range computed from the HGDP-CEPH data set are denoted by *h *and *H*, respectively, then we define the range overlap score (*ROS*) as *z*/*d*, where *d *= ||[*m*, *M*] ∪ [*h*, *H*]|| and *z *= dz* + ||[*m*, *M*] ∩ [*h*, *H*]||(1 - z*). Here z* is the indicator function 1{([*m*, *M*] ⊆ [*h*, *H*]) ∨ ([*m*, *M*] ⊇ [*h*, *H*])}, equaling 1 if the HGDP-CEPH range was a subset of the Marshfield range or *vice versa*, and equaling 0 otherwise. The *ROS *measure was designed for cases in which the two ranges overlapped and neither was contained in the other; if one was contained in the other, then *ROS *reduces to 1 (the notation ||[*m*, *M*]|| for a closed interval refers to the length of the interval, *M *- *m*). A threshold *ROS *value of 0.290 was chosen, as this was the smallest *ROS *observed between the allele size range provided by Marshfield and the allele size range computed from the HGDP-CEPH data set for loci for which the size of the fragment demarcated by a primer pair was both within [*m*, *M*] and within [*h*, *H*]. If a marker had *ROS *≥ 0.290, then it was flagged as "found" if the fragment size was within either the allele size range provided by Marshfield or the allele size range computed from the HGDP-CEPH data set. (iii) As the samples used to define the Marshfield and HGDP-CEPH ranges might not completely capture the full range of human diversity at the loci, it is possible for the RefSeq fragment size to fall just outside the Marshfield and HGDP-CEPH ranges. To account for this possibility, if a marker had *ROS *≥ 0.290, then it was also flagged as "found" if its fragment size was outside both intervals, [*m*, *M*] and [*h*, *H*], but was at most 5 bp outside the allele size range provided by Marshfield or at most 5 bp outside the allele size range computed from the HGDP-CEPH data set.

If the size of the demarcated fragment met one of the three criteria, then its sequence was extracted from the human genome RefSeq database (release 28) in *fasta *format using the standalone *fastacmd *application (version 2.2.18). Only one sequence was extracted per primer pair (microsatellite locus). For one microsatellite locus, D6S942, no allele size range information was available from Marshfield. As the fragment demarcated by its primer pair was within the allele size range computed from the HGDP-CEPH data set, this locus was included in subsequent analyses.

### Analysis of microsatellite sequences

We consider a short tandem repeat (STR) region to be a repeat unit of 2-5 nucleotides with four or more consecutive repeats; a microsatellite locus can contain one or more STR regions embedded between the PCR primers used to amplify the locus. All consecutive repeats of the same repeat unit were considered part of the STR region. A single interruption of one base pair or greater in a run of consecutive repeats of the same repeat unit was considered a break in the repeat structure, and the consecutive runs of repeats on either side of the interruption were considered to be separate STR regions, provided that each run contained at least four repeats.

For each microsatellite the sequence extracted from the human genome RefSeq database (release 28) was interrogated and all STR regions were identified. If more than one STR region was detected, then we determined whether or not the STR regions shared a common repeat unit. The total number of nucleotides separating the embedded STR regions and the total number of repeats at the microsatellite locus were also tabulated. For example, consider a microsatellite locus with three embedded STR regions denoted *A*, *B*, and *C *whose genomic positions have the order *A *<*B *<*C*, whose start and end positions (in base pairs) in the PCR-amplified DNA sequence are denoted by *A*_*start *_and *A*_*end*_, *B*_*start *_and *B*_*end*_, and *C*_*start *_and *C*_*end*_, respectively, and that have *a*, *b*, and c repeats, respectively. The total number of nucleotides separating the embedded STR regions would be given by [(*B*_*start *_- *A*_*end*_) - 1] + [(*C*_*start *_- *B*_*end*_) - 1], and the total number of repeats at the microsatellite locus would be given by *a *+ *b *+ c.

Under the assumption that differences in PCR fragment length are the result of differences in the numbers of repeats in the embedded STR regions, we used the RefSeq sequence of each microsatellite locus to calibrate PCR fragment lengths in individual genotypes to infer repeat number in the HGDP-CEPH data set. At each microsatellite locus, the number of repeats for a PCR fragment length was calculated using *r *+ (*w *- *l*)/*s*, in which *w *is the PCR fragment length (in base pairs), *l *is the length of the sequence in the RefSeq database (in base pairs), *r *is the total number of repeats in the STR regions in the RefSeq sequence, and *s *is the size (in base pairs) of the repeat unit(s) of the STR region(s) embedded in the sequence of the locus (e.g. 4 for a tetra-nucleotide repeat unit). Microsatellite loci with two or more STR regions embedded in their sequence that had repeat units of different sizes were excluded from further analysis as a result of the difficulty in inferring repeat number in the HGDP-CEPH data set. This exclusion made it possible to classify all remaining loci by repeat unit size.

As the context of a repetitive element might be expected to affect its behavior, the flanking sequence of the STR region within three repeat unit lengths of its boundaries (e.g. 12 bp of sequence on either side of an STR region comprised of a tetra-nucleotide repeat unit, a total of 24 nucleotides) was investigated for G/C content. If a boundary of an STR region was within three repeat unit lengths of the end of the extracted RefSeq sequence, then all the sequence between the boundary of the STR region and the end of the extracted RefSeq sequence was considered as flanking sequence. This boundary scenario occurred at the 5' end of the sequence for four microsatellite loci (D7S3065, D12S269, D22S1169, and D22S683) and at the 3' end of the sequence for eleven microsatellite loci (D1S468, D8S1132, D12S1045, D16S539, GATA5E06P, GATA6B07, GATA29C09P, GATA135C03M, GATA152F04M, AGAT132, and NA.D1S.2). Only four of these loci (D1S468, D12S269, D22S1169, and AGAT132) were included for further analysis. The remaining eleven loci were excluded as they each had two or more STR regions embedded in their RefSeq sequence that had repeat units of different sizes. If more than one STR region was embedded in the sequence, then the sequence between each successive pair of STR regions was included in the analysis of the flanking sequence, regardless of length. For the above example of a microsatellite locus with three embedded STR regions denoted *A*, *B*, and *C*, the two regions separating the three embedded STR regions, (*A*_*end*_, *B*_*start*_) and (*B*_*end*_, *C*_*start*_), would be included in the analysis of the flanking sequence along with the sequence regions three repeat unit lengths before *A*_*start *_and three repeat unit lengths after *C*_*end*_.

The G/C content of the flanking sequence was calculated as *y*/*t *where *y *is the number of guanine (G) or cytosine (C) nucleotides within the flanking sequence, and *t *is the total number of nucleotides in the flanking sequence (e.g. 24 for a microsatellite locus with one embedded STR region comprised of a tetra-nucleotide repeat unit).

### Analysis of microsatellite diversity data

Statistical analysis was performed in the R statistical software package (version 2.7.0) [80]. The mean, minimum, maximum, variance, and range of the number of repeats across the 1,048 individuals were calculated from the calibrated HGDP-CEPH data set. The number of distinct alleles and mean fragment size across the 1,048 individuals were calculated from the PCR fragment size data set. The variance (*σ*^2^) in the number of repeats for each microsatellite locus was calculated using the equation , in which *f*_*i *_and *x*_*i *_are the number of observations of allele *i *and the number of repeats in allele *i*, respectively,  is the mean number of repeats, and *k *is the number of distinct alleles.

The skewness in the distribution of the number of repeats (*γ*_1_), potentially reflecting the biases of a microsatellite toward expansion or contraction [81-85], was calculated for each microsatellite locus from the calibrated HGDP-CEPH data set using the *skewness *function (moment method) in the *fBasics *R-package. This function uses equation , in which *g*_*j *_is the number of repeats in observation *j *(among the 2*n *total observations for *n *individuals) and |*σ*| is the standard deviation in the number of repeats. Because *γ*_1 _can be either a positive or negative value, loci with negative values of *γ*_1 _and loci with positive values of *γ*_1 _were considered separately. No loci had *γ*_1 _equal to 0.

Expected heterozygosity (*H*_*e*_) was estimated for each microsatellite locus by treating all 1,048 individuals in the calibrated HGDP-CEPH data set as a single population and using the estimator . In this formula, *n *is the number of individuals (excluding individuals with missing genotype data), *k *is the number of distinct alleles, and  is the relative frequency of allele *i *in the sample. An alternative approach might have involved separately estimating the expected heterozygosity for each locus in each of the 53 populations in the data set, and using the mean heterozygosity across populations for each locus in our analyses. This approach produces values that are highly correlated with those obtained by treating all 1,048 individuals as a single population, as the Pearson product-moment correlation coefficient (*r*) between values obtained by treating all 1,048 individuals as one population and values obtained by taking the mean heterozygosity across all populations was 0.946.

Computation of Spearman's *ρ *correlation coefficient, the Wilxcoxon rank-sum test, and the Kruskal-Wallis test were performed using functions in the *stats *R-package.

## Results

### Microsatellite RefSeq sequence extraction and analysis

**Figure 1:**
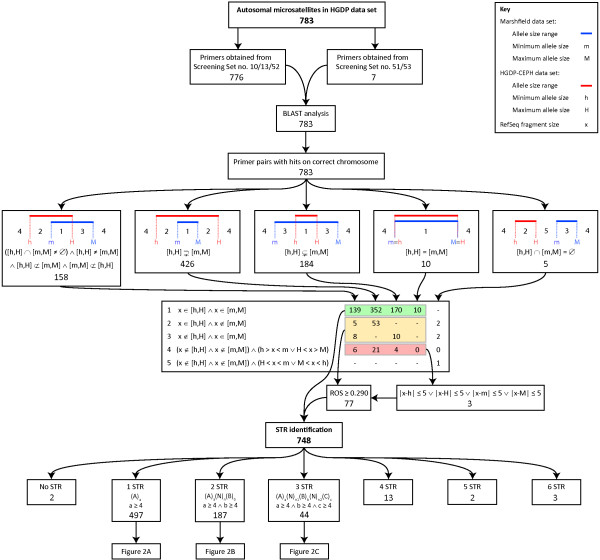
**Summary of the identification and sequence analysis of the microsatellite DNA sequences**. Red bars indicate the allele size range in the HGDP-CEPH data set, for which *h *and *H *are the smallest and largest allele sizes, respectively. Blue bars indicate the allele size range in the Marshfield primer data set, for which *m *and *M *are the smallest and largest allele sizes, respectively. The BLASTN fragment size in the human RefSeq database is denoted by *x*. *A*, *B*, and *C *refer to the repeat units of the different STR regions in a microsatellite sequence, with *a*, *b*, and *c *being the number of times they are repeated, respectively. *N *indicates a nucleotide not within an STR region, with *n *being the number of nucleotides separating two STR regions. For microsatellites with three STR regions, *n*_1 _and *n*_2 _respectively represent the numbers of nucleotides separating the first and second, and the second and third, STR regions. Key: ∧, and; ∨, or; *ROS*, range overlap score.

Primer pairs were successfully obtained for all 783 microsatellite loci, and BLAST analysis of these primer pairs against the human genome RefSeq database (release 28) found that each of the 783 primer pairs identified targets on the correct chromosome for its associated locus. Of the 783 microsatellite loci, 748 (95.5%) were retained for further analysis (Figure 1). Five loci were excluded because the allele size range provided by Marshfield and the allele size range computed from the HGDP-CEPH data set did not overlap. Twenty-eight loci were excluded because the size of the fragment identified in the RefSeq database was more than 5 bp outside the allele size range provided by Marshfield and more than 5 bp outside the allele size range computed from the HGDP-CEPH data set. Two loci (D8S262 and GAAT1F09P) were excluded because their RefSeq fragment sizes were outside the allele size ranges provided by Marshfield and their *ROS *values of 0.133 and 0.263, respectively, were below the specified threshold of 0.290.

**Figure 2:**
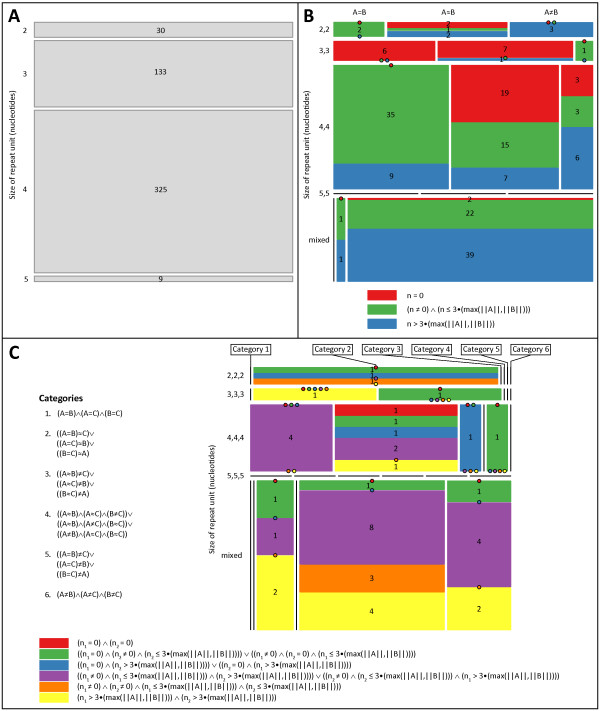
**Mosaic plots describing microsatellites with (A) one, (B) two or (C) three separate STR regions**. In the mosaic plots [117,118], tiles represent categories of microsatellites, and the area of each tile is proportional to the number of microsatellites in the corresponding category. For loci with two or more STR regions, loci are first grouped by the relationships between the repeat units in their STR regions - identical ("*A*=*B*"), similar ("*A*≈*B*"; 1 bp difference between their sequences), or different by more than 1 bp ("*A*≠*B*") - and by the sizes of those repeat units (i.e. di-, tri-, tetra-, or penta-nucleotide, with "mixed" referring to loci whose STR regions are comprised of repeat units of different sizes). Each group of loci is partitioned into distinct categories based on the distance (in nucleotides) separating their STR regions, as described below the plot, and each category is represented in a different color. Black bars represent groups that contain no loci. Filled circles represent those categories within a group that contain no loci. For microsatellites with two STR regions, *n *represents the number of nucleotides separating the first and second STR regions. For microsatellites with three STR regions, *n*_1 _and *n*_2 _respectively represent the numbers of nucleotides separating the first and second, and the second and third, STR regions. Key: ∧, and; ∨, or; ||*A*||, length of *A*.

The repeat structure of each of the 748 remaining microsatellite loci was investigated, and short tandem repeat (STR) regions were identified (Figure 2). Loci with one STR region embedded in their sequence with a di- (30), tri- (133), or a tetra- (325) nucleotide repeat unit, and loci with two (10, 15, and 97, respectively) or three (3, 2, and 12, respectively) separate STR regions whose repeat units had the same size were retained for further analysis. The 65 and 27 loci with two or three separate STR regions, respectively, whose repeat units had different sizes, were excluded because of the resulting difficulty in assigning repeat number in the HGDP-CEPH data set. The nine loci with a single STR region comprised of a penta-nucleotide repeat unit and the 18 loci with four or more STR regions embedded in their sequence were excluded because of small sample size. Two additional loci (AAT267 and AAT249) were excluded because no STR regions were identified within their extracted RefSeq sequence. Therefore, of the original 783 microsatellite loci, 627 (80.1%) were retained for the population-genetic analysis (Figures 1 and 2). For each of the 627 microsatellite loci used in the population-genetic analysis, the primer sequences, extracted human RefSeq sequence, and repeat structure identified within that sequence can be found in Table S1 (Additional File 1), and the values calculated for all variables can be found in Table S2 (Additional File 2).

For 390 of the 627 microsatellite loci used in the population-genetic analysis, all allele sizes in the HGDP-CEPH were separated by exact multiples of the size of their repeat unit (Table 1). These loci are termed "regular." The remaining 237 loci were found to possess one or more alleles whose sizes were not separated from their flanking alleles by exact multiples of the size of their repeat unit ("irregular"). In most cases, ~2/3 of the loci in locus classifications with a tri- or tetra-nucleotide repeat unit were "regular," and the remaining ~1/3 of the loci were "irregular" (Table 1). For di-nucleotide loci, the corresponding fractions were ~4/5 "regular" loci and ~1/5 "irregular" loci.

**Table 1:** The number of microsatellite loci with regular and irregular allele structure

	Regular	Irregular
	
Number of loci	390	237
One STR region	302	186

Di	25	5
Tri	94	39
Tetra	183	142

Two separate STR regions	76	46

Di	8	2
Tri	10	5
Tetra	58	39

Three separate STR regions	12	5

Di	3	0
Tri	1	1
Tetra	8	4

### Relationship of microsatellite sequence properties and microsatellite heterozygosity

#### Chromosome

Microsatellite heterozygosity did not vary significantly across chromosomes in separate tests for di-, tri-, or tetra-nucleotide microsatellite loci with one, two, or three separate STR regions (*P *> 0.05 in all comparisons, Kruskal-Wallis test), nor did any of the microsatellite sequence properties or measures of variation across individuals (Table S3, Kruskal-Wallis tests; Additional File 3). Note, however, that many of the Kruskal-Wallis tests would have had little power to detect a difference across chromosomes, as a consequence of small ratios of the number of loci to the number of chromosomes.

#### Repeat unit size

In agreement with several previous reports [73,86-88], we found that heterozygosity was inversely related to the size of the STR region's repeat unit in microsatellite loci with a single embedded STR region (*P *= 0.005, Kruskal-Wallis test; Table 2). Loci with a di-nucleotide repeat unit had higher heterozygosity than loci with a tri-nucleotide repeat unit (*P *= 0.027, Wilcoxon test); di-nucleotide loci also had higher heterozygosity than loci with a tetra-nucleotide repeat unit (*P *= 0.002, Wilcoxon test), as did loci with a tri-nucleotide repeat unit (*P *= 0.046, Wilcoxon test). However, the inverse relationship between repeat unit size and heterozygosity was not observed in microsatellite loci with two separate STR regions (Table 2). Although loci with two separate di-nucleotide STR regions had higher heterozygosity than loci with two separate tri-nucleotide STR regions (*P *= 0.041, Wilcoxon test), the latter were found to have lower heterozygosity than loci with two separate tetra-nucleotide STR regions (*P *= 0.064, Wilcoxon test). However, fewer loci were examined in these comparisons than in comparisons involving only one STR region.

**Table 2:** The effect of repeat unit size on microsatellite heterozygosity

	**1 STR region**			**2 STR regions**		
		
	Di	Tri	Tetra	Di	Tri	Tetra
Number of loci	30	133	325	10	15	97
	
Mean *H*_*e*_	0.779	0.749	0.739	0.789	0.721	0.772
	
Di		**0.027**	**0.002**		**0.041**	0.246
Tri			**0.046**			0.064

*P *values are shown for two-sided Wilcoxon rank-sum tests for differences in heterozygosity (*H*_*e*_) between microsatellites with different repeat unit sizes. Loci are grouped by whether they had one or two separate STR regions. No comparisons were made for loci with three separate STR regions because of small sample size for di-nucleotide and tri-nucleotide loci (3 and 2, respectively). Tests with *P *< 0.05 are highlighted in **bold**.

#### Repeat unit sequence

Among microsatellite loci with a single embedded tetra-nucleotide STR region, the sequence of the repeat unit was found to have a significant effect on microsatellite heterozygosity (*P *= 0.026, Kruskal-Wallis test). This overall effect is visible in pairwise comparisons of different tetra-nucleotide repeat unit sequences (Table 3). Loci with repeat unit AAGG or its reverse complement TTCC were found to have significantly higher heterozygosity than loci with repeat unit AAAT or its reverse complement TTTA (*P *= 4.80 × 10^-5^, Wilcoxon test), as were loci with repeat unit ATCT or its reverse complement TAGA (*P *= 4.49 × 10^-4^, Wilcoxon test). In general, we observed a trend between increases in the number of guanine (G) and cytosine (C) nucleotides in the repeat unit sequence and increases in heterozygosity. A comparison between the heterozygosities of the 30 tetra-nucleotide loci with no G/C nucleotides in their repeat unit sequence (mean *H*_*e *_= 0.683), the 268 tetra-nucleotide loci with one G/C nucleotide in their repeat unit sequence (mean *H*_*e *_= 0.742), and the 23 tetra-nucleotide loci with two G/C nucleotides in their repeat unit sequence (mean *H*_*e *_= 0.775) found that loci with one or two G/C nucleotides had significantly higher heterozygosity than those with no G/C nucleotides (*P *= 6.48 × 10^-4 ^and *P *= 2.15 × 10^-4^, respectively, Wilcoxon test). A similar comparison between tetra-nucleotide loci with one or two G/C nucleotides in their repeat unit sequence found that those loci with two G/C nucleotides had significantly higher heterozygosity than loci with one G/C nucleotide (*P *= 0.025, Wilcoxon test).

**Table 3:** The effect of repeat unit sequence on microsatellite heterozygosity for tetra-nucleotide loci

	AAAT & TTTA	AATG	CATA & GTAT	TTCA	GATG	ATCT & TAGA	AAGG & TTCC
Number of loci	30	4	8	3	5	253	18
Mean *H*_*e*_	0.683	0.691	0.714	0.719	0.722	0.744	0.789

AAAT [AATA-ATAA-TAAA] & TTTA [TTAT-TATT-ATTT]		0.979	0.407	0.416	0.421	**4.49 × 10^-4^**	**4.80 × 10^-5^**
AATG [ATGA-TGAA-GAAT]			0.808	0.857	0.556	0.229	0.066
CATA [ATAC-TACA-ACAT] & GTAT [TATG-ATGT-TGTA]				0.921	1	0.239	**0.011**
TTCA [TCAT-CATT-ATTC]					0.786	0.541	0.080
GATG [ATGG-TGGA-GGAT]						0.304	**0.019**
ATCT [TCTA-CTAT-TATC] & TAGA [AGAT-GATA-ATAG]							**0.003**
AAGG [AGGA-GGAA-GAAG] & TTCC [TCCT-CCTT-CTTC]							

*P *values are shown for two-sided Wilcoxon rank-sum tests for differences in heterozygosity (*H*_*e*_) between the different repeat unit sequences of microsatellites with one tetra-nucleotide STR region. Four loci were excluded from these comparisons because their repeat unit sequences only appeared once (TTTG and GAAA) or twice (TCCA) in the data set. Tests with *P *< 0.05 are highlighted in **bold**.

Only two repeat unit sequences were observed for microsatellite loci with a single embedded di-nucleotide STR region (AC and GT) and only three loci possessed GT repeats. Six repeat unit sequences were observed among microsatellite loci with a single embedded tri-nucleotide STR region (AAT, TTA, ATC, ATG, CTG, and AAC). However, only loci with repeat units AAT and TTA appeared more than twice in our data set. Heterozygosity was not significantly different between the 70 tri-nucleotide loci with repeat unit AAT and the 57 loci with repeat unit TTA (*P *= 0.279, Wilcoxon test). The lack of a significant difference in heterozygosity in this comparison possibly reflects the relationship of these repeat units as reverse complement sequences of one another. Similarly, no significant difference in heterozygosity was observed between the 12 loci with a single embedded tetra-nucleotide STR region and repeat unit AAGG (mean *H*_*e *_= 0.796) and the six loci with repeat unit CCTT (mean *H*_*e *_= 0.777), the reverse complement of AAGG (*P *= 0.494, Wilcoxon test), between the 124 loci with repeat unit ATCT (mean *H*_*e *_= 0.737) and the 129 loci with repeat unit TAGA (mean *H*_*e *_= 0.751), the reverse complement of ATCT (*P *= 0.093, Wilcoxon test), between the 15 loci with a single embedded tetra-nucleotide STR region and repeat unit AAAT (mean *H*_*e *_= 0.656) and the 15 loci with repeat unit TTTA (mean *H*_*e *_= 0.710), the reverse complement of AAAT (*P *= 0.106, Wilcoxon test), or between the three loci with repeat unit ATAC (mean *H*_*e *_= 0.692) and the five loci with repeat unit TATG (mean *H*_*e *_= 0.727), the reverse complement of ATAC (*P *= 0.786, Wilcoxon test). These results support the explanation that for loci with a single embedded tri-nucleotide STR region, no significant difference in heterozygosity was observed between loci with the two different repeat unit sequences because of the reverse complementary relationship of the two sequence motifs.

#### Number of distinct STR regions

The number of separate tetra-nucleotide STR regions in a microsatellite sequence was found to significantly increase the heterozygosity of the microsatellite (Table 4). Loci with one STR region had lower heterozygosity than those with two (*P *= 3.12 × 10^-5^, Wilcoxon test) or three (*P *= 4.23 × 10^-4^, Wilcoxon test) separate STR regions. Additionally, loci with two separate STR regions had lower heterozygosity than those with three separate STR regions (*P *= 0.049, Wilcoxon test). The number of separate STR regions in loci with a di- or tri-nucleotide repeat unit, for which fewer loci were examined, did not significantly affect heterozygosity (Table 4).

**Table 4:** The effect of the number of separate STR regions on microsatellite heterozygosity

	**Di**		**Tri**		**Tetra**		
			
	1 STR	2 STRs	1 STR	2 STRs	1 STR	2 STRs	3 STRs
Number of loci	30	10	133	15	325	97	12
		
Mean *H*_*e*_	0.779	0.789	0.749	0.721	0.739	0.772	0.814
		
1 STR region	0.770			0.319		**3.12 × 10^-5^**	**4.23 × 10^-4^**
STR regions							**0.049**

*P *values are shown for two-sided Wilcoxon rank-sum tests for differences in heterozygosity (*H*_*e*_) between microsatellites with one, two, or three separate STR regions. Loci are grouped by their repeat unit size. For di-nucleotide and tri-nucleotide loci, because of small sample size (3 and 2, respectively), the column for three separate STR regions was omitted. Tests with *P *< 0.05 are highlighted in **bold**.

#### All identical versus not all identical repeat units

For microsatellite loci with two or more separate STR regions embedded in their sequence, the identity or non-identity of their repeat units was not found to influence their heterozygosity. The seven tri-nucleotide microsatellite loci with two separate STR regions and identical repeat units did not have significantly different heterozygosity from the eight loci that had non-identical repeat units (*P *= 0.281, Wilcoxon test). Additionally, the 50 tetra-nucleotide microsatellite loci with two separate STR regions and identical repeat units did not differ significantly in heterozygosity from the 47 that had non-identical repeat units (*P *= 0.621, Wilcoxon test), and the five tetra-nucleotide microsatellite loci with three separate STR regions and identical repeat units did not differ significantly in heterozygosity from the seven that had nonidentical repeat units (*P *= 0.343, Wilcoxon test).

#### Distance separating distinct STR regions

The number of nucleotides separating two separate STR regions with a tri-nucleotide repeat unit was found to be positively correlated with heterozygosity (*ρ *= 0.568, *P *= 0.027). However, for di-nucleotide repeat units the number of nucleotides separating two separate STR regions was not significantly correlated with heterozygosity at the *P *= 0.05 level (Table 5). Similarly, for tetra-nucleotide repeat units the total number of nucleotides separating two or three separate STR regions was not significantly correlated with heterozygosity (Table 5).

**Table 5:** Spearman's rank correlations of heterozygosity with microsatellite sequence properties and measures of variation across individuals

	1 STR region			2 STR regions			3 STR regions
			
	Di	Tri	Tetra	Di	Tri	Tetra	Tetra
Number of loci	30	133	325	10	15	97	12
**Sequence properties**							
G/C content of flanking sequence	0.247	0.037	-0.034	0.237	0.291	0.142	0.035
Number of nucleotides separating STR regions	-	-	-	0.140	**0.568**	0.032	0.252
**Measures of variation across individuals**							
Number of distinct alleles	**0.424**	**0.431**	**0.517**	0.628	0.497	**0.596**	**0.644**
Variance in number of repeats	0.325	**0.502**	**0.800**	**0.636**	**0.750**	**0.779**	**0.846**
Range of number of repeats	0.151	**0.326**	**0.492**	**0.646**	0.304	**0.672**	**0.681**
Mean PCR fragment size	0.040	0.007	-0.016	-0152	**0.571**	0.126	0.308
Mean number of repeats	**0.564**	**0.239**	**0.134**	0.455	0.018	**0.388**	0.503
Maximum number of repeats	**0.465**	**0.228**	**0.384**	**0.669**	0.235	**0.562**	**0.706**
Minimum number of repeats	0.335	-0.036	-0.044	0.049	-0.079	-0.036	0.385

Spearman's rank correlation coefficients (*ρ*) are shown for comparisons of microsatellite heterozygosity with continuous microsatellite sequence properties and with measures of variation across individuals in the HGDP-CEPH data set. Microsatellites were classified by the number of separate STR regions embedded in their sequence and by their repeat unit size. Hyphens indicate comparisons that were not evaluated. For three STR regions, no comparisons were performed for di-nucleotide and tri-nucleotide loci because of small sample size (3 and 2, respectively). Correlations with *P *< 0.05 are highlighted in **bold**.

#### G/C content of sequence flanking STR regions

In agreement with previous studies [70,89], the G/C content of the sequence flanking the STR region of microsatellite loci with a single embedded di-nucleotide STR region was not strongly correlated with microsatellite heterozygosity (*ρ *= 0.247, *P *= 0.188). The G/C content of the sequence flanking the STR region of loci with a single embedded tri- or tetra-nucleotide STR region was also not significantly correlated with heterozygosity (Table 5). Similarly, we found no significant correlation between G/C content of the sequence flanking the STR regions and heterozygosity for di-, tri-, and tetra-nucleotide loci with two separate STR regions, or for tetra-nucleotide loci with three separate STR regions (Table 5).

### Relationship of microsatellite population properties and microsatellite heterozygosity

A summary of the mean, minimum, and maximum values across loci for microsatellite population properties appears in Table S4 (Additional File 4).

#### Number of distinct alleles

The number of distinct alleles at tetra-nucleotide microsatellite loci with one (*ρ *= 0.517, *P *= 1.34 × 10^-23^), two (*ρ *= 0.596, *P *= 1.17 × 10^-10^), or three (*ρ *= 0.644, *P *= 0.024) separate STR regions was positively correlated with microsatellite heterozygosity. Similarly, the number of distinct alleles at di- (*ρ *= 0.424, *P *= 0.019) and tri-nucleotide (*ρ *= 0.431, *P *= 2.31 × 10^-7^) loci with one STR region embedded in their sequence was positively correlated with heterozygosity. However, the number of distinct alleles was not significantly correlated with heterozygosity for di- and tri-nucleotide loci with two separate STR regions (Table 5).

#### Variance in the number of repeats

We found that variance in the number of repeats was positively correlated with microsatellite heterozygosity (Table 5) for tri- and tetra-nucleotide microsatellite loci with one (*ρ *= 0.502 with *P *= 7.45 × 10^-10^, and *ρ *= 0.800 with *P *= 1.42 × 10^-73^, respectively) or two (*ρ *= 0.750 with *P *= 1.28 × 10^-3^, and *ρ *= 0.779 with *P *= 6.03 × 10^-21^, respectively) separate STR regions, and for tetra-nucleotide loci with three separate STR regions (*ρ *= 0.846, *P *= 5.21 × 10^-4^). Similarly, variance in the number of repeats was positively correlated with heterozygosity for di-nucleotide loci with two separate STR regions (*ρ *= 0.636, *P *= 0.048). However, no significant correlation between variance in the number of repeats and heterozygosity was detected for di-nucleotide loci with a single STR region embedded in their sequence (Table 5).

#### Range of the number of repeats

The range of the number of repeats was positively correlated with microsatellite heterozygosity (Table 5) for tetra-nucleotide microsatellite loci with one (*ρ *= 0.492, *P *= 3.44 × 10^-21^), two (*ρ *= 0.672, *P *= 4.62 × 10^-14^), or three (*ρ *= 0.681, *P *= 0.015) separate STR regions. The range of the number of repeats was also positively correlated with heterozygosity for tri-nucleotide microsatellite loci with only one STR region embedded in their sequence (*ρ *= 0.326, *P *= 1.26 × 10^-4^) and for di-nucleotide microsatellite loci with two separate STR regions (*ρ *= 0.646, *P *= 0.044). However, there was no significant correlation between the range of the number of repeats and heterozygosity for di-nucleotide microsatellite loci with one STR region, or for tri-nucleotide loci with two separate STR regions (Table 5).

#### Skewness in the distribution of the number of repeats

The skewness in the distribution of the number of repeats (*γ*_1_) was negatively correlated with microsatellite heterozygosity for microsatellite loci with a single tri- or tetra-nucleotide STR region embedded in their sequence (Table 6). Considering only microsatellite loci that had negative *γ*_1_, the heterozygosities of the 73 loci with a single tri-nucleotide STR region (*ρ *= -0.333, *P *= 0.004) and the 201 loci with a single tetra-nucleotide STR region (*ρ *= -0.291, *P *= 2.81 × 10^-5^) were negatively correlated with *γ*_1_. However, if only loci that had positive *γ*_1 _were considered, the same negative correlation between *γ*_1 _and heterozygosity was found for the 60 microsatellite loci with a single tri-nucleotide STR region (*ρ *= -0.545, *P *= 6.83 × 10^-6^), but not for the 124 loci with a single tetra-nucleotide STR region (*ρ *= -0.043, *P *= 0.633). Similarly, no significant correlation was found between *γ*_1 _and heterozygosity for the 42 loci with two separate tetra-nucleotide STR regions and positive *γ*_1 _(*ρ *= -0.225, *P *= 0.152) or for the 55 loci with two separate tetra-nucleotide STR regions and negative *γ*_1 _(*ρ *= -0.124, *P *= 0.368). Additionally, no significant correlation was found between *γ*_1 _and heterozygosity for the 12 loci with a single di-nucleotide STR region and negative *γ*_1 _(*ρ *= -0.105, *P *= 0.746) or for the 18 loci with a single di-nucleotide STR region and positive *γ*_1 _(*ρ *= -0.007, *P *= 0.977).

**Table 6:** Spearman's rank correlations of heterozygosity with skewness in the number of repeats across individuals

	**1 STR region**			**2 STR regions**			**3 STR regions**
			
	**Di**	**Tri**	**Tetra**	**Di**	**Tri**	**Tetra**	**Tetra**
		
Number of loci	12	**73**	**201**	7	12	55	4
*γ*_1 _< 0	-0.105	**-0.333**	**-0.291**	-0.286	-0.252	-0.124	-
Number of loci	18	**60**	124	3	3	42	8
*γ*_1 _> 0	-0.007	**-0.545**	-0.043	-	-	-0.225	-0.143

Spearman's rank correlation coefficients (*ρ*) are shown for comparisons of microsatellite heterozygosity with skewness in the distribution of the number of repeats across individuals in the HGDP-CEPH data set. Microsatellites were classified by the number of separate STR regions embedded in their sequence, by their repeat unit size, and based on whether skewness (*γ*_1_) was greater or less than zero. Hyphens indicate comparisons that were not evaluated. For three STR regions, no comparisons were performed for di-nucleotide and tri-nucleotide loci because of small sample size (3 and 2, respectively). Correlations with *P *< 0.05 are highlighted in **bold**.

#### Mean PCR fragment length

In agreement with a previous report [90], we found no significant correlation between microsatellite heterozygosity and mean PCR fragment length for di-nucleotide microsatellite loci with one STR region embedded in their sequence (*ρ *= 0.040, *P *= 0.835). We also found no significant correlation between heterozygosity and mean PCR fragment length for tri- and tetra-nucleotide loci with one STR region embedded in their sequence (Table 5). Similarly, we found no significant correlation between heterozygosity and mean PCR fragment length for di-, and tetra-nucleotide loci with two separate STR regions. However, a significant correlation was found for tri-nucleotide loci with two separate STR regions (*ρ *= 0.571, *P *= 0.026).

#### Mean of the number of repeats

In agreement with a previous report in *Drosophila melanogaster *[70], we found the mean number of repeats to be positively correlated with microsatellite heterozygosity for microsatellite loci with one embedded di-nucleotide STR region (*ρ *= 0.564, *P *= 1.16 × 10^-3^). The mean number of repeats was also positively correlated with heterozygosity for loci with one embedded tri- (*ρ *= 0.239, *P *= 0.006) or tetra- (*ρ *= 0.134, *P *= 0.015) nucleotide STR region. Similarly, the mean number of repeats was positively correlated with heterozygosity for tetra-nucleotide loci with two separate STR regions (*ρ *= 0.388, *P *= 8.45 × 10^-5^). However, no significant correlation was found between the mean number of repeats and heterozygosity for di- or tri-nucleotide loci with two separate STR regions, or for tetra-nucleotide loci with three separate STR regions (Table 5).

#### Minimum and maximum number of repeats

In agreement with a previous report [70], we found the maximum number of repeats to be positively correlated with microsatellite heterozygosity for loci with a single embedded di-nucleotide STR region (*ρ *= 0.465, *P *= 0.010). We also found the maximum number of repeats to be positively correlated with heterozygosity for loci with two separate di-nucleotide STR regions (*ρ *= 0.669, *P *= 0.035). The maximum number of repeats was also positively correlated with heterozygosity for tetra-nucleotide loci with one (*ρ *= 0.384, *P *= 7.38 × 10^-13^), two (*ρ *= 0.562, *P *= 2.18 × 10^-9^), or three (*ρ *= 0.706, *P *= 0.010) separate STR regions. Similarly, the maximum number of repeats was positively correlated with heterozygosity for loci with a single embedded tri-nucleotide STR region (*ρ *= 0.228, *P *= 0.008). However, no significant correlation was observed between the maximum number of repeats and heterozygosity for tri-nucleotide loci with two separate STR regions (Table 5). Additionally, no significant correlation was observed between microsatellite heterozygosity and the minimum number of repeats for di-, tri-, or tetra-nucleotide loci with one or two separate STR regions, or for tetra-nucleotide loci with three separate STR regions (Table 5).

## Discussion

Our study provides the most comprehensive evaluation to date of the effect of sequence properties of microsatellites on microsatellite variability in human populations. The relatively large number of microsatellites examined here has enabled us to consider the relationships with microsatellite heterozygosity of a wide variety of sequence properties.

Our results confirm the well-known relationship between the size of the repeat unit of a microsatellite locus and the variability of the locus [73,86,87], with larger repeat units leading to lower heterozygosity (Table 2). In agreement with this trend, smaller repeat unit size was also found to lead to a higher mean number of repeats, and we observed that a higher mean number of repeats led to higher heterozygosity (Table 5). For microsatellites with a single embedded STR region, loci with a di-nucleotide repeat unit had higher mean numbers of repeats (mean = 18.16) than loci with a tri-nucleotide repeat unit (mean = 13.79; *P *= 2.14 × 10^-12^, Wilcoxon test) and loci with a tetra-nucleotide repeat unit (mean = 12.03; *P *< 10^-15^, Wilcoxon test); loci with a tri-nucleotide repeat unit also had higher mean numbers of repeats than loci with a tetra-nucleotide repeat unit (*P *= 3.33 × 10^-15^, Wilcoxon test). Previous studies comparing loci with the same number of repeats but different repeat unit sizes reported the same trend that larger repeat unit size led to lower microsatellite variability [88,91], suggesting that our observed relationship between repeat unit size and heterozygosity is not wholly due to the correlations of both quantities with the mean number of repeats.

We also found the composition of the repeat unit of tetra-nucleotide microsatellite loci to be an important factor in predicting heterozygosity, with repeat units high in G/C content leading to higher heterozygosity. This result agrees with a previous study [70] that reported that of the three most common di-nucleotide repeat units in *Drosophila melanogaster *(TC/AG, AT/TA, and GT/CA), microsatellite loci with repeat units GT/CA and TC/GA had higher mutation rates than loci with repeat unit AT/TA. It also agrees with the observations of a comparative genomics study of three unrelated chicken individuals [92] that reported that tri-nucleotide repeat units high in G/C content had higher variability than tri-nucleotide repeat units low in G/C content. However, it is important to note that our results might be specific to the particular motifs available in our data set. We have only one motif that contains no G/C nucleotides (AAAT/TTTA) and only two motifs that contain two G/C nucleotides (GATG/CTAC and AAGG/TTCC), and together these motifs represent only ~1/6 of the tetra-nucleotide loci we examined (30 loci have no G/C nucleotides in their repeat motif and 23 loci have two G/C nucleotides in their repeat motif). Additionally, of the remaining 268 tetra-nucleotide loci, 253 contain the same repeat unit (ATCT/TAGA).

Our observed correlation between increases in the G/C content of the repeat unit of tetra-nucleotide microsatellite loci and increases in heterozygosity disagrees with a comparative genomics study that found that tetra-nucleotide repeat units high in G/C content led to lower variability in chickens [92]. It also disagrees with the findings of a second comparative genomics study of human and chimpanzee orthologous tetra-nucleotide microsatellite loci that detected no significant correlation between repeat unit composition and the average squared difference in the number of repeats between orthologs [91]. The two comparative genomics studies differ from ours in considering many more loci, but using many fewer individuals for estimating population diversity. Thus, differences in results between our study and the comparative genomics studies could arise because neither of the comparative genomics studies is entirely analogous to ours: Brandstrom and Ellegren [92] considered data from only a small number of individuals compared to our analysis of 1,048 human individuals, and the approach taken by Kelkar *et al*. [91] is quite different from ours in being focused on genomes of different species. It is also possible that a difference arose from ascertainment of highly polymorphic loci in the genotyping panels used in our study compared to the relatively bias-free approach offered by comparative genomics. However, we have no reason to suspect that a marker ascertainment procedure selecting for variability would have produced a systematic difference in variability between different motifs. It is also possible that loci in our study might have experienced a greater degree of natural selection compared to the genome as a whole. However, a previous report by Kayser *et al*. [93] on 332 microsatellite loci with considerable overlap with the loci in our study found that natural selection did influence the vast majority of the loci. Investigating scores of the iHS test for natural selection, calculated from SNP genotype data in the three Phase I and II HapMap populations [94] in 100-Kb regions centered on each microsatellite locus we consider here, we find that almost all loci lie within regions that have mean iHS scores that were not considered significant by Voight *et al*. (mean iHS in CEU = 0.018, minimum = -1.048, maximum = 1.270; mean iHS in YRI = 0.034, minimum = -0.996, maximum = 1.797; mean iHS in ASN = 0.022, minimum = -1.331, maximum = 1.244). Thus, natural selection is not likely to have strongly influenced our results.

Our results regarding the effect of repeat unit composition on microsatellite variability also disagree with the results of Eckert *et al*. [95], who reported that tetra-nucleotide loci with one G/C nucleotide in their repeat unit (AGAT/TCTA and AAAG/TTTC) exhibited higher mutation rates than those with two G/C nucleotides (AAGG/TTCC). However, in our data (Table 3), loci with repeat unit ATCT/TAGA (referred to as AGAT/TCTA by Eckert *et al*. [95]) had significantly lower heterozygosity than loci with repeat unit AAGG/TTCC (*P *= 0.003, Wilcoxon test), suggesting that the differences between our results and those of Eckert *et al*. [95] are not necessarily a consequence of differences in the sequence composition of the repeat units. Our data set was obtained by genotyping 1,048 individuals for each of the 325 tetra-nucleotide loci whereas Eckert *et al*. [95] used vector-based arrays of repeats in a human B lymphoblastoid cell line. The differences between the two studies could therefore be the result of distinct cellular environments between the two studies, as our study considers accumulations of germline mutations, whereas somatic mutations were considered by Eckert *et al*. [95]. Additionally, the DNA environments differ, as we consider genomic DNA whereas Eckert *et al*. [95] examined reporter constructs.

We found tetra-nucleotide microsatellite loci containing more separate sets of repeated motifs to have generally higher heterozygosity. This observation disagrees with two previous reports that found uninterrupted arrays of *Drosophila melanogaster *di- and tri-nucleotide repeats [72] and human di-nucleotide repeats [1] to be more polymorphic than those that had interruptions. It also disagrees with studies of vector-based poly-GT arrays in *Saccharomyces cerevisiae *[96] and poly-CTG arrays in a human astrocyte cell line [97] that similarly reported that interruptions in the array of repeats led to decreased variability. An important difference between our study and some of those previously reported is our inclusion of interrupted loci whose STR regions were separated by arbitrary lengths. We also applied a different threshold when defining runs of repeats, requiring four or more repeats before we considered a run of repeats as an STR region, whereas Weber [1], for example, required three or more repeats, and Goldstein and Clark [72] required two. Another difference between our study and that of Goldstein and Clark [72] is that we used the total number of repeats across all STR regions at a locus, whereas their correlations with variance considered only the number of repeats in the longest run of repeats. The differences between our study and previous studies could therefore result from differences in experimental design. It is also possible that the correlation we observed between more separate sets of repeated motifs and higher heterozygosity applies to human tetra-nucleotide loci but not to other scenarios considered by previous studies.

In agreement with a previous study [90], PCR fragment size was found to have no correlation with microsatellite variability. This is unsurprising given that PCR primer pairs are positioned so as to optimize the amplification of the locus, and their locations do not have intrinsic biological meaning. Because the distance from embedded STR regions will vary among PCR primer pairs, PCR fragment sizes do not represent absolute numbers of repeats and therefore are not comparable in a meaningful way between different loci. When we converted PCR fragment sizes into underlying numbers of repeats, however, we did find that the mean number of repeats across individuals was positively correlated with heterozygosity. Similarly, we found the maximum number of repeats across individuals to be positively correlated with heterozygosity. Some of these observations might arise from a general correlation among the various measures of diversity (Tables S5 and S6; see Additional File 5 and Additional File 6, respectively); they are consistent with previous reports in *Drosophila melanogaster *[70,72,73] that found the mean and maximum number of repeats to be positively correlated with the variability of di- and tri-nucleotide microsatellite loci, and with reports in humans [31,34] that found the mean number of repeats to be positively correlated with mutation rate of tetra-nucleotide loci. They also agree with studies that reported that increases in the length of the repetitive component of the sequence, measured in base pairs [84,98-100] or number of repeats [91,92], led to higher rates of mutation [84,99,100], polymorphism [92,98], and average squared differences in the number of repeats between orthologous loci [91].

The correlations we have observed between heterozygosity and the size and sequence of the repeat unit and the mean and maximum number of repeats are concordant with those reported between microsatellite mutation rate and repeat unit size [73,86], mutation rate and repeat unit sequence [70], and mutation rate and microsatellite length [34,101,102]. The most commonly proposed mutation mechanism for microsatellites is replication slippage [4,103]; because of homology among microsatellite repeats, the two DNA strands might realign incorrectly after polymerase dissociation and strand separation, introducing a loop in one strand and leading to microsatellite expansion or contraction after the resumption of replication [104]. How then can our observed correlations between the sequence properties of microsatellites and heterozygosity be explained in terms of their relationship to the mutation mechanism?

The direct relationship between heterozygosity and the number of distinct STR regions and the direct relationship between heterozygosity and measures reflecting microsatellite length (mean and maximum number of repeats) might very well reflect increases in the probability of slippage as a function of the number of repeats at which it can occur [84,91,105]. Similarly, the inverse relationship between heterozygosity and repeat unit length might reflect the increased probability of incorrect realignment after the dissociation of two DNA strands comprised of small repeated motifs compared to those comprised of large repeated motifs. For a given microsatellite length measured in nucleotides, twice as many di-nucleotide repeat units would exist compared to tetra-nucleotide repeat units, with the number of tri-nucleotide repeat units being intermediate between those of di- and tetra-nucleotide repeat units. During strand realignment, di-nucleotide repeat units would therefore have a greater chance of mispairing than both tri- and tetra-nucleotide repeat units, because of the larger number of repeated motifs present in the disassociated DNA strands; tri-nucleotide repeat units would similarly have a greater chance of mispairing than tetra-nucleotide repeat units.

Because slippage involves the loss and reforming of hydrogen bonds [106], the influence of the sequence composition of the (tetra-nucleotide) repeat motif on heterozygosity, in which higher G/C content led to higher heterozygosity, might be attributable to the higher number of hydrogen bonds in the double-stranded DNA offered by G/C pairs that stabilize the mispaired intermediate after DNA strand dissociation and reannealing. For example, repeat unit AAGG would form 10 hydrogen bonds (two per A/T base pair and three per G/C base pair) compared to the 8 hydrogen bonds formed by repeat unit AAAT. The two additional hydrogen bonds in mispaired AAGG intermediates compared with mispaired AAAT intermediates would be expected to provide increased stability, potentially enabling more of the mispaired AAGG intermediates than mispaired AAAT intermediates to remain paired until the resumption of strand synthesis. However, with this reasoning, we would expect that the weaker hydrogen bonds for A/T pairs would cause paired strands rich in A/T nucleotides to dissociate more frequently than paired strands rich in G/C nucleotides, providing more opportunities for A/T rich sequences to undergo slippage-induced mutations. If hydrogen bonding is an important determinant of mutability, then the observation that motifs rich in G/C nucleotides lead to higher variability suggests that the effect of G/C nucleotides in stabilizing mispaired intermediates exceeds that of A/T nucleotides in generating more opportunities for mutation. Alternatively, we note that various studies have suggested mechanisms by which certain motifs might produce more mutation than others [103,107-112], and it is possible that our observation of an effect of G/C content on variability is an artifact of a more general effect of motif composition on variability.

In conclusion, considerations of mechanisms of microsatellite mutation suggest a view in which those microsatellite sequence properties that we have observed to influence heterozygosity do so by altering the chance that a mutation event will occur. Within this perspective, increased repeat unit size acts to reduce the chance that a mutation event occurs, thereby reducing heterozygosity; increases in the number of G/C nucleotides in the repeat unit, the number of distinct STR regions, and measures of microsatellite length (mean and maximum number of repeats) all act to increase the chance that a mutation event occurs, thereby increasing heterozygosity.

## Conclusion

By jointly considering sequence properties of microsatellites in the human RefSeq sequence together with properties of genetic diversity in human populations, we have produced the first genome-wide systematic analysis of the relationship between diverse microsatellite sequence properties and features of human microsatellite variability. However, it is important to note that we have not sequenced the microsatellites in each individual and have instead assumed that differences in PCR fragment length reflect differences in numbers of copies of embedded repeat units. Further, these microsatellite loci, which we used because they had been previously studied in a worldwide collection of individuals, might not be representative of all human microsatellites. We have no reason to suspect that either of these issues might have systematically affected the particular comparisons that we have performed. For future work, however, comparative genomics with multiple human genome sequences offers a relatively bias-free approach for the random or comprehensive sampling of microsatellite loci when applied to the genome sequences of many individuals of the same species. Current short-read next-generation sequencing platforms [113,114] are ill-equipped to interrogate long runs of repetitive sequences such as microsatellites that can cover several hundred base pairs of DNA. As the longer read lengths expected for "third generation" sequencing platforms [115,116] offer sequence reads capable of interrogating repetitive sequences, the resequencing of many human individuals will allow for a more detailed examination of how the sequence properties of microsatellites affect their variability in human populations.

## Abbreviations

*ROS*: range overlap score; STR: short tandem repeats; HGDP: Human Genome Diversity Project; CEPH: Centre d'Etude du Polymorphisme Humain.

## Competing interests

The authors declare that they have no competing interests.

## Authors' contributions

NAR, MJ, and TJP conceived the study. TJP, CIS, and MJ performed the analysis. TJP and NAR wrote the paper. All authors read and approved the final manuscript.

## Supplementary Material

Additional file 1 **Table S1**. The primer sequences, extracted human RefSeq sequence, and the repeat structure identified within that sequence (demarcated by square brackets in the RefSeq sequence), for each of the 627 microsatellite loci used in the population-genetic analysis. Click here for file

Additional file 2 **Table S2**. The variables calculated for each of the 627 microsatellite loci used in the population-genetic analysis. Click here for file

Additional file 3 **Table S3**. The effect of chromosome number on the different sequence properties and measures of variation across individuals. Click here for file

Additional file 4 **Table S4**. Summary of the properties of measures of variation across individuals. Click here for file

Additional file 5 **Table S5**. Spearman's rank correlations between measures of variation across individuals for microsatellites with one or two separate STR regions embedded in their sequence. Click here for file

Additional file 6 **Table S6**. Spearman's rank correlations between measures of variation across individuals for microsatellites with three separate tetra-nucleotide STR regions embedded in their sequence. Click here for file
