# Supplementary material for: Sequence determinants of human microsatellite variability
Source: BMC Genomics. 2009 Dec 16;10:612. doi: 10.1186/1471-2164-10-612 (PMC2806349; doi:10.1186/1471-2164-10-612)
Supplement: Additional file 3 — Table S3. The effect of chromosome number on the different sequence properties and measures of variation across individuals. [file 1471-2164-10-612-S3.PDF]

Table S3. The effect of chromosome number on microsatellite sequence properties and measures of variation across individuals

		1 STR region			2 STR regions			3 STR regions
		Di	Tri	Tetra	Di	Tri	Tetra	Tetra
	Number of loci	30	133	325	10	15	97	12
Sequence properties	G/C content of STR region(s) flanking sequence	0.556	0.064	0.428	0.361	0.650	0.058	0.347
	Number of nucleotides separating distinct STR regions	-	-	-	0.403	0.382	0.514	0.515
Measures of variation across individuals	Heterozygosity	0.864	0.362	0.756	0.347	0.598	0.520	0.625
	Number of distinct alleles	0.581	0.668	0.932	0.353	0.860	0.450	0.485
	Variance in number of repeats	0.448	0.206	0.921	0.347	0.537	0.678	0.515
	Range of number of repeats	0.487	0.895	0.896	0.362	0.801	0.786	0.471
	Skewness in number of repeats	0.166	0.853	0.532	0.347	0.710	0.343	0.332
	Mean PCR fragment length	0.489	0.323	0.996	0.361	0.508	0.748	0.323
	Mean number of repeats	0.564	0.403	0.525	0.347	0.372	0.727	0.550
	Maximum number of repeats	0.314	0.731	0.647	0.347	0.230	0.549	0.637
	Minimum number of repeats	0.509	0.552	0.784	0.386	0.408	0.389	0.563

P values from Kruskal-Wallis tests for differences in microsatellite heterozygosity, sequence properties, and measures of variation across individuals in the HGDP-CEPH data set when grouped by chromosome number. Microsatellites were classified by the number of separate STR regions embedded in their sequence and by their repeat unit size. Loci with three separate di-nucleotide or tri-nucleotide STR regions were not evaluated due to small sample size (3 and 2, respectively). Hyphens indicate comparisons that were not evaluated.
