# Supplementary material for: Sequence determinants of human microsatellite variability
Source: BMC Genomics. 2009 Dec 16;10:612. doi: 10.1186/1471-2164-10-612 (PMC2806349; doi:10.1186/1471-2164-10-612)
Supplement: Additional file 4 — Table S4. Summary of the properties of measures of variation across individuals. [file 1471-2164-10-612-S4.PDF]

Table S4. Summary of the properties of the measures of variation across individuals

		1 STR region			2 STR Regions			3 STR regions
		Di	Tri	Tetra	Di	Tri	Tetra	Tetra
Number of loci		30	133	325	10	15	97	12
Number of repeats in RefSeq	Mean	17.73	12.68	11.20	22.10	15.93	16.31	22.42
	Minimum	4	6	4	16	12	10	17
	Maximum	27	17	23	30	21	27	32
Heterozygosity	Mean	0.779	0.749	0.739	0.789	0.721	0.772	0.814
	Minimum	0.606	0.313	0.507	0.614	0.526	0.568	0.675
	Maximum	0.882	0.867	0.907	0.855	0.843	0.908	0.895
Number of distinct alleles	Mean	15.37	11.47	10.86	14.30	10.67	12.59	14.83
	Minimum	9	5	5	6	4	7	9
	Maximum	24	23	29	21	20	35	28
Variance in number of repeats	Mean	8.53	3.57	2.39	5.61	3.06	3.87	4.21
	Minimum	1.41	0.36	0.49	1.87	0.77	0.59	1.17
	Maximum	29.79	13.77	24.62	17.51	6.93	33.92	8.11
Range of number of repeats	Mean	16.13	9.85	9.13	14.40	9.29	10.79	13.13
	Minimum	10	4	4	5	3	5	8
	Maximum	24	19	23	23	12	24	20
Skewness in number of repeats	Mean	0.067	-0.060	-0.177	-0.015	-0.531	-0.057	0.191
	Minimum	-0.921	-2.053	-2.649	-1.182	-1.728	-1.900	-0.459
	Maximum	1.153	2.661	2.425	2.234	0.648	2.804	0.974
Mean PCR fragment size	Mean	158.56	190.21	201.90	181.54	204.68	218.26	241.34
	Minimum	98.30	94.32	102.44	118.28	144.30	115.52	148.27
	Maximum	257.77	319.15	412.49	232.10	273.66	413.16	355.83
Mean number of repeats	Mean	18.16	13.79	12.03	23.72	17.58	16.91	21.63
	Minimum	13.36	9.19	5.18	17.83	12.25	9.46	17.66
	Maximum	23.72	18.94	22.30	31.05	23.00	27.20	32.27
Maximum number of repeats	Mean	26.57	18.80	16.52	31.50	21.84	22.28	28.31
	Minimum	21	12.67	8.75	21	17	13.25	21.50
	Maximum	34	26.67	30.75	43	27	33.50	42.25
Minimum number of repeats	Mean	10.43	8.95	7.39	17.10	12.56	11.49	15.19
	Minimum	6	3	-0.25*	12	6.67	3.50	8
	Maximum	17	14.33	13.25	24	19	20.50	22.25

Microsatellites were classified by the number of separate STR regions embedded in their sequence and by their repeat unit size. For three STR regions, no values are presented for di-nucleotide and tri-nucleotide loci because of small sample size (3 and 2, respectively).

*This negative value was obtained with the smallest allele at the tetra-nucleotide locus AGAT113Z (Table S2), present in only a single individual in our data set. The fragment identified for this locus in RefSeq was 185 bp and contained a single STR region with 7 repeats (Table S1). The smallest allele identified for this locus in our data set was 156 bp. However, the size expected for an allele containing 0 repeats is 157 bp based on the identified RefSeq fragment. There are at least two likely scenarios under which this size anomaly may have occurred: (1) The 156 bp allele contains 0 repeats but because of a -1 bp shift in the PCR fragment size during genotyping it appears to have a negative non-integer number of repeats. (2) A deletion could have occurred in the non-repetitive part of the region between the two primers, creating a PCR fragment smaller than expected for reasons unrelated to the properties of the repetitive element.
