# Supplementary material for: Sequence determinants of human microsatellite variability
Source: BMC Genomics. 2009 Dec 16;10:612. doi: 10.1186/1471-2164-10-612 (PMC2806349; doi:10.1186/1471-2164-10-612)
Supplement: Additional file 5 — Table S5. Spearman's rank correlations between measures of variation across individuals for microsatellites with one or two separate STR regions embedded in their sequence. [file 1471-2164-10-612-S5.PDF]

Table S5. Spearman's rank correlations between measures of variation across individuals for microsatellites with one or two STR regions embedded in their sequence

		Number of distinct alleles		Variance in number of repeats		Range of number of repeats		Skewness in number of repeats		Mean PCR fragment size		Mean number of repeats		Maximum number of repeats		Minimum number of repeats	
		ρ	*P*	ρ	*P*	ρ	*P*	ρ	*P*	ρ	*P*	ρ	*P*	ρ	*P*	ρ	*P*
Number of distinct alleles	Di	-	-	**0.651**	**9.88x10^{-5}**	**0.735**	**3.74x10^{-6}**	0.338	0.067	-0.006	0.975	0.286	0.126	**0.765**	**8.37x10^{-7}**	-0.002	0.991
	Tri	-	-	**0.375**	**8.94x10^{-6}**	**0.696**	**1.46x10^{-20}**	-0.131	0.133	0.085	0.329	**0.247**	**0.004**	**0.400**	**1.81x10^{-6}**	-0.143	0.101
	Tetra	-	-	**0.513**	**3.14x10^{-23}**	**0.825**	**3.48x10^{-82}**	0.027	0.625	0.052	0.348	**0.258**	**2.38x10^{-6}**	**0.575**	**5.22x10^{-30}**	**-0.110**	**0.047**
Variance in number of repeats	Di	0.494	0.147	-	-	**0.657**	**8.08x10^{-5}**	0.004	0.984	0.004	0.984	0.014	0.940	**0.616**	**2.89x10^{-4}**	-0.071	0.709
	Tri	**0.713**	**0.003**	-	-	**0.415**	**6.64x10^{-7}**	-0.142	0.104	0.058	0.506	-0.029	0.740	**0.231**	**0.008**	-0.130	0.135
	Tetra	**0.674**	**3.75x10^{-14}**	-	-	**0.549**	**4.97x10^{-27}**	**0.127**	**0.022**	0.009	0.873	0.007	0.904	**0.300**	**3.38x10^{-8}**	**-0.205**	**1.99x10^{-4}**
Range of number of repeats	Di	**0.985**	**2.44x10^{-7}**	0.394	0.260	-	-	0.405	0.026	0	0.998	0.067	0.726	**0.628**	**2.00x10^{-4}**	**-0.505**	**0.004**
	Tri	**0.893**	**7.59x10^{-6}**	**0.679**	**0.005**	-	-	-0.096	0.270	0.033	0.706	**0.239**	**0.006**	**0.501**	**7.84x10^{-10}**	**-0.281**	**1.06x10^{-3}**
	Tetra	**0.838**	**9.71x10^{-27}**	**0.771**	**2.52x10^{-20}**	-	-	0.108	0.053	0.071	0.203	**0.288**	**1.24x10^{-7}**	**0.650**	**2.23x10^{-40}**	**-0.183**	**8.90x10^{-4}**
Skewness in number of repeats	Di	0.207	0.565	-0.139	0.701	0.172	0.634	-	-	-0.043	0.820	-0.094	0.623	0.153	0.420	**-0.432**	**0.017**
	Tri	-0.148	0.599	-0.161	0.567	0.114	0.686	-	-	0.104	0.232	0.002	0.983	-0.041	0.639	0.084	0.334
	Tetra	**0.216**	**0.034**	0.163	0.111	0.157	0.124	-	-	0.016	0.779	-0.079	0.156	-0.052	0.347	**-0.164**	**0.003**
Mean PCR fragment size	Di	0.439	0.204	-0.103	0.777	0.437	0.207	0.309	0.385	-	-	0.114	0.550	0.020	0.916	-0.002	0.991
	Tri	-0.042	0.882	0.321	0.243	-0.147	0.602	-0.254	0.362	-	-	0.072	0.410	0.062	0.477	0.029	0.742
	Tetra	0.127	0.216	-0.005	0.964	0.120	0.241	0.031	0.765	-	-	0.052	0.348	0.045	0.424	-0.036	0.521
Mean number of repeats	Di	**0.866**	**1.20x10^{-3}**	0.455	0.187	**0.825**	**0.003**	-0.018	0.960	0.394	0.260	-	-	**0.656**	**8.20x10^{-5}**	**0.597**	**5.03x10^{-4}**
	Tri	-0.115	0.683	-0.104	0.713	-0.163	0.562	0.229	0.413	-0.279	0.315	-	-	**0.838**	**3.40x10^{-36}**	**0.737**	**4.77x10^{-24}**
	Tetra	**0.294**	**0.004**	**0.239**	**0.019**	**0.391**	**7.43x10^{-5}**	-0.015	0.884	**0.319**	**1.47x10^{-3}**	-	-	**0.817**	**2.47x10^{-79}**	**0.753**	**1.17x10^{-60}**
Maximum number of repeats	Di	**0.945**	**3.76x10^{-5}**	0.559	0.093	**0.914**	**2.19x10^{-4}**	-0.024	0.947	0.413	0.235	**0.912**	**2.37x10^{-4}**	-	-	0.322	0.083
	Tri	0.149	0.597	0.185	0.509	0.026	0.926	-0.059	0.834	-0.072	0.799	**0.876**	**1.85x10^{-5}**	-	-	**0.642**	**7.75x10^{-17}**
	Tetra	**0.617**	**1.65x10^{-11}**	**0.546**	**7.11x10^{-9}**	**0.725**	**4.65x10^{-17}**	0.100	0.328	**0.251**	**0.013**	**0.836**	**1.75x10^{-26}**	-	-	**0.549**	**5.23x10^{-27}**
Minimum number of repeats	Di	0.352	0.319	0.280	0.434	0.265	0.459	-0.292	0.413	0.413	0.235	0.596	0.069	0.573	0.083	-	-
	Tri	-0.351	0.200	-0.259	0.352	-0.507	0.054	-0.052	0.854	-0.115	0.683	**0.785**	**5.33x10^{-4}**	**0.726**	**0.002**	-	-
	Tetra	-0.169	0.099	-0.140	0.171	-0.199	0.051	-0.093	0.366	**0.231**	**0.023**	**0.677**	**2.65x10^{-14}**	**0.445**	**4.96x10^{-6}**	-	-

Spearman's rank correlation coefficients (ρ) and their associated *P* values are shown for comparisons of the measures of variation across individuals in the HGDP-CEPH data set for loci with one (upper triangle) or two (lower triangle) STR regions embedded in their sequence. $|\gamma_1|$, the absolute value of skewness γ_1, was used in evaluating correlations with skewness in number of repeats. Microsatellites were classified by the number of separate STR regions embedded in their sequence and by their repeat unit size. Sample sizes for loci with one STR region were 30, 133, and 325 for di-nucleotides, tri-nucleotides, and tetra-nucleotides, respectively, and for loci with two separate STR regions they were 11, 15, and 97, respectively. Correlations with $P < 0.05$ are highlighted in **bold**.
