# Supplementary material for: Sequence determinants of human microsatellite variability
Source: BMC Genomics. 2009 Dec 16;10:612. doi: 10.1186/1471-2164-10-612 (PMC2806349; doi:10.1186/1471-2164-10-612)
Supplement: Additional file 6 — Table S6. Spearman's rank correlations between measures of variation across individuals for microsatellites with three separate tetra-nucleotide STR regions embedded in their sequence. [file 1471-2164-10-612-S6.PDF]

Table S6. Spearman's rank correlations between measures of variation across individuals for microsatellites with three tetra-nucleotide STR regions embedded in their sequence

	Variance in number of repeats		Range of number of repeats		Skewness in number of repeats		Mean PCR fragment size		Mean number of repeats		Maximum number of repeats		Minimum number of repeats	
	ρ	*P*	ρ	*P*	ρ	*P*	ρ	*P*	ρ	*P*	ρ	*P*	ρ	*P*
Number of distinct alleles	**0.848**	**$4.98x10^{-4}$**	**0.979**	**$3.24x10^{-8}$**	0.336	0.285	0.308	0.330	**0.602**	**0.038**	**0.771**	**0.003**	0.312	0.323
Variance in number of repeats	-	-	**0.843**	**$5.72x10^{-4}$**	0.259	0.417	0.203	0.527	0.350	0.265	**0.629**	**0.028**	0.070	0.829
Range of number of repeats			-	-	0.317	0.315	0.275	0.387	**0.646**	**0.023**	**0.825**	**$9.45x10^{-4}$**	0.343	0.275
Skewness in number of repeats					-	-	-0.357	0.255	0.084	0.795	0.280	0.379	0.042	0.897
Mean PCR fragment size							-	-	0.238	0.457	0.301	0.342	0.175	0.586
Mean number of repeats									-	-	**0.881**	**$1.53x10^{-4}$**	**0.862**	**$3.15x10^{-4}$**
Maximum number of repeats											-	-	**0.687**	**0.014**
Minimum number of repeats													-	-

Spearman's rank correlation coefficients (ρ) and their associated *P* values are shown for comparisons of the measures of variation across individuals in the HGDP-CEPH data set for the 12 loci with three tetra-nucleotide STR regions embedded in their sequence. No comparisons were performed for di-nucleotide and tri-nucleotide loci with three STR regions embedded in their sequence because of small sample size (3 and 2, respectively). Correlations with $P < 0.05$ are highlighted in **bold**.
